# Design of Urban resilience model in emergency situations

**DOI:** 10.1371/journal.pone.0347588

**Published:** 2026-06-09

**Authors:** Dongxun Jiang, Zhaocheng Li

**Affiliations:** 1 Guohao College, Tongji University, Shanghai, China; 2 School of Mathematical Science, Tongji University, Shanghai, China; Sun Yat-Sen University, CHINA

## Abstract

In response to the current demand for urban resilience in emergency situations, we have developed an urban resilience assessment system for responding to sudden natural disasters based on housing data, infrastructure data, as well as Gross Domestic Product(GDP) and population data from 25 regions in Changchun and Hohhot. Subsequently, we conducted multidimensional consideration and analysis of the infrastructure service levels of the two cities, and based on this, designed aurora resilience in emergency situations. We have introduced three evaluation indicators: emergency response speed, resource allocation efficiency, and per capita facility density. We use Analytic Hierarchy Process (AHP) to analyze importance weights and obtain robustness of comprehensive infrastructure. Based on this, we used TOPSIS method to analyze the problems faced by two cities at different service levels and provided long-term and short-term investment strategies. Finally, we discussed the advantages and disadvantages of current urban resilience assessment models and conducted consistency and sensitivity analysis on the AHP algorithm. We found that existing models can to some extent reflect the stability and resilience of cities.

## 1 Introduction

Recently, the escalating frequency and intensity of extreme weather events have placed unprecedented strain on urban infrastructure networks, housing safety systems, and transportation arteries. Nowadays, the specter of climate disasters looms globally, profoundly threatening urban living stability and compelling us to re-examine survival strategies for urban systems in crisis. At this critical juncture, constructing resilient cities—complex systems that maintain or rapidly recover to ideal states when encountering disturbances and possess long-term adaptive capacity—has transcended academic discourse to become a core agenda for global policymakers pursuing sustainable prosperity [1–3].

Faced with the immense pressure that extreme weather poses on urban development, enhancing urban resilience—the comprehensive capacity to resist internal and external shocks, safeguard resident safety, and promote sustainable development—has become a key benchmark for measuring urban strength [4–6]. Although this has become a closely watched issue, existing research still faces profound challenges in systematic assessment and supporting practical decision-making. Early explorations mostly focused on the robustness of single infrastructures: Beheshtian et al. [7] quantified the impact of climate extreme events on New York’s transportation-energy systems, Gulsrud et al. [8] revealed the role of Melbourne’s “green place” strategy in promoting socio-ecological adaptation, while Levenda et al. [9] analyzed the flow and mutation of smart grid policies across multiple cities. Despite their pioneering nature, these studies are universally constrained by the geographic boundaries of single cases and static data—making cross-regional comparisons difficult and failing to incorporate economic costs and resource allocation efficiency into core considerations. Tablada and Zhao et al. [10] explored the relationship between tropical urban density and resource self-sufficiency with forward-looking vision, yet similarly failed to translate into immediate policy tools due to the lack of real-time dynamic data. Therefore, how to transcend the infrastructure perspective and advance from multi-dimensional physical assessment to executable, budgetable action plans has become a critical challenge.

In recent years, the research perspective on urban resilience has gradually deepened toward the micro-physical environment and adaptive performance of individual buildings, driving a leap in assessment precision. De Masi et al. [11] revealed the risk of failure of traditional energy-saving measures under global warming, Gonzales and Ajami et al. [12] proposed a bottom-up water resource resilience framework, while assessments targeting livelihood support in informal settlements [13] and the potential of floating production systems [14] continuously expand theoretical boundaries. Technically, Baniassadi and Sailor et al. [15] confirmed that improving building air tightness can significantly enhance passive survival capabilities during extreme heat and power outage events; Kastner and Dogan et al. [16] developed the Eddy3D simulation tool, which dramatically improved annual outdoor thermal comfort assessment efficiency by decoupling urban wind environment and radiation calculations; Laetitia et al. [17] coupled dynamic energy consumption models with CFD fluid simulation, providing precise strategies for natural ventilation retrofitting of high-rise slab buildings in hot-summer-cold-winter regions. Soltani and Sharifi et al. [18] further confirmed using mobile observation technology that green coverage rate (especially within 500-meter buffers) shows a significant negative correlation with nighttime urban heat island intensity. However, despite substantial methodological sophistication, over seventy percent of studies still concentrate on coastal developed megacities [19], while urban resilience issues faced by northern cities like Changchun and Hohhot have not been effectively assessed and addressed. More critically, these studies mostly rely on time-lagged population censuses or single physical field simulations, making it difficult to capture real-time dynamics of urban functions and lacking horizontal comparability due to inconsistent data standards, ultimately failing to integrate functional dimensions that best reflect resilience [20–22]. A core dilemma thus emerges: although existing assessment systems can diagnose hardware shortcomings, they cannot establish quantifiable conversion bridges between “strategic assessment” and “fiscal investment.”

To construct a comprehensive framework that can both reflect real-time functional status and guide budget allocation, we employ Point of Interest (POI) data for integrated urban resilience assessment. Compared with traditional data, POI offers unprecedented possibilities for capturing urban dynamics through characteristics of real-time updates, building-scale spatial precision, and flexible customization. In recent years, people have utilized POI for multidimensional exploration [23–28], and these explorations have highlighted the wide applicability of POI. However, there is still a problem with these studies: current applications mostly remain at the descriptive statistical level of “single-hazard, single-function,” generating popularity rankings through subjective weighting, neither coupling with economic parameters nor answering the core policy concern of “where should investment be prioritized under limited budgets.” In short, we possess more refined data but still have not established an automated transformation mechanism from “indicator calculation” to “action plans.”

To address the aforementioned gaps, this study constructs a three-dimensional “distance-density-economy” matrix assessment framework and innovatively integrates the Analytic Hierarchy Process (AHP) with the Technique for Order Preference by Similarity to Ideal Solution (TOPSIS), achieving full-process connectivity from diagnosis to decision-making. This framework not only integrates POI data for 15 categories of urban service facilities but also introduces economic signals such as housing prices and parking fees as adjustment factors, enabling interactive effects between facility spatial distribution and cost affordability within the same vector. Through AHP, expert experience is transformed into verifiable weights, and TOPSIS subsequently linearly maps abstract resilience scores to fiscal gap ratios, thereby generating specific investment quotas in units of hundred million yuan. This design converts traditionally difficult-to-quantify social, economic, and physical dimensions in assessment into budgetary language directly accessible for municipal finance departments.

Compared with existing research, the contributions of this paper can be summarized into four points:

1. Proposing a cross-regionally transferable three-dimensional “distance-density-economy” assessment framework that, for the first time, deeply integrates 15 categories of POI facilities with economic data such as housing prices and GDP, breaking through single-dimension, single-hazard assessment limitations.
2. Adopting an AHP-TOPSIS hybrid method that balances expert knowledge embedding with data-driven ranking under low sample size conditions (25 districts/counties), providing a methodological paradigm for similar small-to-medium sample policy assessments.
3. Remedying the “assessment-heavy, implementation-light” deficiency in existing research by directly transforming model outputs into phased, facility-specific investment plan tables, connecting three major policy tools: budget preparation, land use planning, and infrastructure procurement.
4. Enriching the geographical scope of climate resilience research by focusing on the two northern cities of Changchun and Hohhot, providing a replicable assessment template for similar resource-constrained cities.

## 2 Related work

### 2.1 Urban resilience and multi-scenario applications based on POI data

In recent years, research on urban resilience based on Point of Interest (POI) data has made significant progress across multiple fields. For instance, one study utilized multi-source social media data—including geotagged Twitter and Flickr data—to investigate the resilience of urban vitality in inner-city London during the COVID-19 pandemic. It assessed the resilience capabilities of different POI types, such as transportation hubs, educational and healthcare institutions, restaurants, and financial centers, demonstratinag how these areas adapted to change [23]. Another study focused on the distribution and accessibility of urban green spaces in Yangzhou, China. By employing POI data specific to green spaces, it highlighted disparities in green space accessibility between urban and suburban areas, recommending targeted urban planning interventions to enhance resilience [24]. Additionally, research on community resilience during Hurricane Harvey in Houston employed digital footprint data linked to unique visit POIs. By quantifying resilience metrics based on human mobility fluctuations across different POIs, it revealed insights into systemic impacts, duration of effects, and overall resilience values [25].

Concurrently, POI data has played a significant role in flood recovery rates, natural gas pipeline risk assessments, and traffic collision risk analysis. For instance, a study assessing urban flood resilience in the Wei River basin during Henan’s rainstorms utilized POI data to evaluate county-level flood recovery rates (FRR). Results indicated that POIs significantly influenced FRR and positively impacted recovery levels [26]. In urban natural gas pipeline risk research, integrating POI data constructed a socio-spatial distribution model for potential pipeline accidents. By combining physical and social vulnerabilities, it provided comprehensive risk assessments to enhance urban resilience [27]. A Shanghai-based study combined POI data with nighttime light intensity data to generate collision risk maps for vehicle-pedestrian and vehicle-vehicle incidents, demonstrating POI data’s potential in predicting diverse road traffic collision risks [28]. These studies indicate that POI data holds broad application prospects in urban resilience assessments, providing scientific foundations for urban planning and emergency management.

Existing studies predominantly treat POIs as “single-hazard, single-function” counting metrics, subjectively assigning weights to generate popularity rankings. This approach lacks integration with economic parameters and fails to address the question of “where to prioritize investments with limited budgets.” We integrate all fifteen POI categories into a three-dimensional “distance-density-economic” matrix. Using AHP, we derive scenario-specific weights for emergency response. Then, TOPSIS converts each district’s distance from the ideal solution into a proportional investment gap. Finally, we allocate short- and long-term investments proportionally, generating a detailed list of facilities and investment quotas in hundreds of millions of yuan. This approach fills the gap between having metrics and having actionable solutions.

### 2.2 Key dimensions and classic indicators in current Urban resilience studies

To understand the key components and parameters traditionally used to measure urban resilience, we can draw from various academic sources that provide comprehensive frameworks and indicators. The main components of urban resilience include social, economic, environmental, physical, and institutional dimensions.

Social resilience is a critical dimension that encompasses education and awareness, public health services, and community support. Enhancing knowledge and awareness among residents is essential for building a resilient community [29,30]. Robust public health services and social well-being are also crucial components of social resilience [31]. Strengthening community networks and support systems further enhances the ability of communities to withstand and recover from disturbances [29].

Economic resilience focuses on economic diversity, employment and income stability, and strategies for rapid economic recovery. Promoting diverse economic activities reduces dependency on a single sector, thereby enhancing economic resilience [31]. Ensuring stable employment opportunities and equitable income distribution is vital for maintaining economic stability [30,32]. Developing strategies for rapid economic recovery post-disaster is another key aspect of economic resilience [31].

Environmental resilience involves green infrastructure, climate adaptation, and natural resource management. Implementing green spaces and sustainable land use practices contributes to environmental resilience [33,34]. Developing strategies to adapt to climate change impacts is essential for cities facing environmental challenges [33,35]. Efficient management of natural resources also supports overall resilience [36].

Physical resilience includes infrastructure robustness, strategic urban planning, and resilient transportation systems. Ensuring the robustness of buildings and infrastructure is fundamental for withstanding physical disturbances [37]. Strategic urban planning aims to reduce vulnerability and enhance adaptive capacities [38,39]. Developing resilient transportation systems is crucial for maintaining mobility during disruptions [40].

Institutional resilience focuses on governance and leadership, policy and regulation, and stakeholder collaboration. Strong leadership and governance structures are essential for managing resilience efforts effectively [35,41]. Implementing policies that support resilience and disaster risk reduction is another key aspect [35,42]. Encouraging collaboration among various stakeholders, including government, the private sector, and communities, enhances the overall effectiveness of resilience strategies [41,42].

In addition to these components, various parameters and indicators are used to measure urban resilience. The Complex Resilience Index is a composite indicator that includes social, economic, and environmental components to measure overall resilience [32]. The Climate Disaster Resilience Index (CDRI) incorporates parameters such as physical, social, economic, institutional, and natural factors [42]. The Urban Resilience Index measures diversity, self-sufficiency, polycentric governance, social cohesion, learning and innovation, and social-ecological justice [35,43]. The Key City Resilience Indicators (KCRI) cluster indicators to facilitate the measurement of urban resilience across different contexts [44].

These indicators can be applied in various ways to assess different aspects of urban resilience. For example, parking fees can be used to assess economic resilience by evaluating the affordability and accessibility of urban areas, which impacts economic activities and mobility [40]. Housing prices reflect economic stability and social resilience. While high housing prices may indicate economic prosperity, they can also highlight social inequality and affordability issues, which are critical for assessing social resilience [31,32].

In conclusion, the integration of these indicators into the urban resilience framework helps create a comprehensive understanding of a city’s ability to withstand and recover from various disturbances. By focusing on these key components and parameters, urban planners and policymakers can develop more effective strategies to enhance a city’s resilience.

Traditional frameworks emphasize five pillars: social, economic, environmental, physical, and institutional. However, implementation often stalls due to challenges in quantifying indicators, reaching consensus on weightings, and directing investment based on outcomes. In our framework, taking housing prices and parking fees as examples, we design them as a dual “economic-physical” factor: housing price deviation reflects affordability, while parking price differential reflects transportation structure. Together, they adjust the per capita facility density score, enabling price signals and spatial signals to interact within the same vector. After AHP-TOPSIS integration, districts with high housing prices and low facility density automatically slide toward a “negative ideal solution,” securing higher investment priority. This approach translates the abstract goals of the five pillars into executable, budgetable, and trackable expressions.

## 3 Methods

### 3.1 Data preprocessing and data analysis

Firstly, We collected data including GDP and population of cities and regions, as well as related housing prices, urban infrastructure POI, etc. from the 58.com and the official websites of relevant government departments. Due to some missing data, For quantifiable data types, we will use the mean or median instead of missing values based on actual situations. For data types that can only be analyzed qualitatively, the model will use more reasonable methods to fill in missing values. For example, if the building type is missing, in the subsequent feature preprocessing stage, the model uses MultiLabelBinarizer for multi label binary encoding, filling in the missing values as “other.” At this point, the missing values will not affect the encoding results.

**Fig 1:**
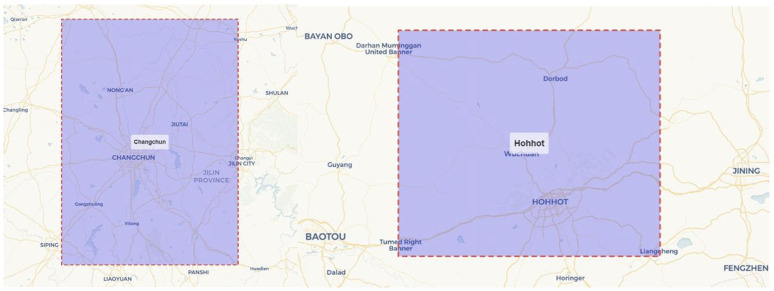
Rough outline range of Changchun (left)/Hohhot (right). Map tiles by CARTO, © OpenStreetMap contributors, © CARTO. Map data © OpenStreetMap contributors, available under the Open Database License (ODbL) (https://www.openstreetmap.org/copyright). Map created using Leaflet (https://leafletjs.com). The rectangular boundaries indicate the approximate study areas for data collection.

Meanwhile, by observing the longitude (lon) and latitude (lat) columns, we found several significant deviations in the longitude coordinates of Hohhot. Considering the authenticity of the coordinates, we replaced these deviation values with the average coordinates. On this basis, we separately calculated the maximum and minimum values of the latitude and longitude coordinates of two cities, and circled the approximate ranges of Changchun and Hohhot with rectangles on the map of China, as shown in Fig 1.

**Fig 2:**
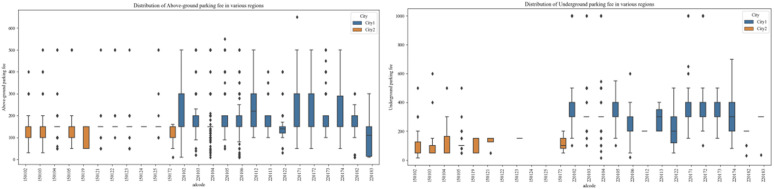
Box plot of above ground parking fee (left)/underground parking fee (right) distribution in different regions.

**Fig 3:**
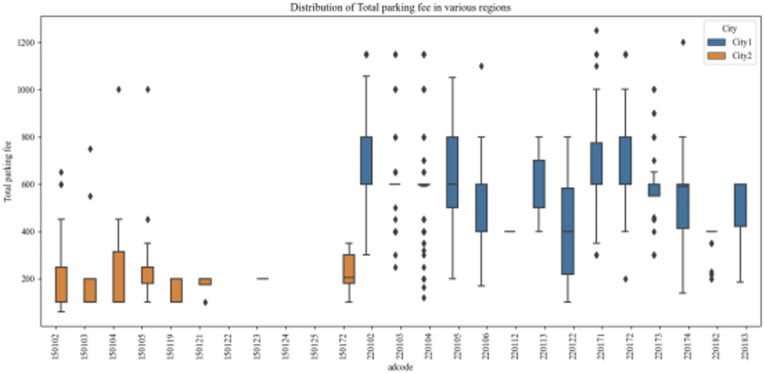
Box plot of Total Parking Fee distribution in different regions.

Afterwards, we conducted relevant statistics and analysis on the existing data, obtained the characteristics and correlations between the existing data, and thus drew box plots of different data types or building indicators in different regions. By observing and comparing these icons, we found that certain data may exhibit positive or negative correlation trends, and may also be associated with certain indicators. For example, from Figs 2 and 3, we can see that there may be a certain positive correlation between underground parking fee and total parking fee. However, areas with high above ground parking fee may have lower total parking fee and do not have a very obvious correlation. Therefore, we infer that parking in both cities may be mainly underground parking, and above ground parking space may be scarce. Therefore, areas with higher above ground parking fees may also have higher housing prices.

**Fig 4:**
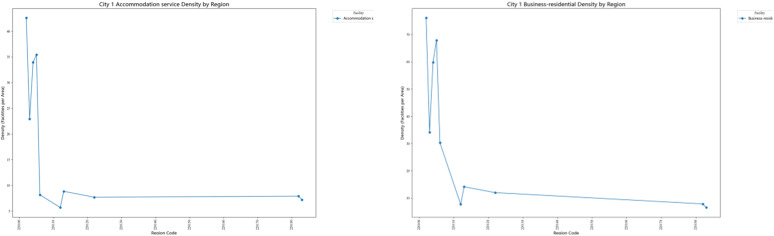
Line graph of density distribution of different facilities in different areas.

Similarly, we can analyze multiple images to infer factors that may affect housing prices and urban resilience, then, based on the obtained data, the categories and distribution of different service facilities in Changchun and Hohhot were listed separately in Fig 4. And we have drawn line charts of the density distribution of different facilities in different areas, which can more intuitively show the density of related facilities in different areas.

Before introducing our method, let’s make the following assumptions:

The location information mentioned in this article is based on latitude and longitude as reference coordinates.
In this article, the total number of households is used instead of the total population for valuation calculation (in fact, if each household has an average of a people, then the total population = total number of households * a, that is, the per capita result = average household result * a, where a is not a fixed value and is determined by the local population situation).
Assuming that the real estate market is not affected by force majeure factors such as major policy changes or economic crises during the forecast period.
Assuming that the service facilities mentioned can meet all people’s needs and reflect the local ability to respond to extreme weather and emergency situations.
Assuming that extreme weather events occur independently, without considering the impact of consecutive events.
6. Assuming that all service resources in the city are available and efficiently allocated when dealing with extreme weather and emergency situations.

### 3.2 Quantitative analysis of urban service level

If we want to conduct a comprehensive analysis of the service levels of two cities, we need to consider multiple factors.

Firstly, we consider the richness of different types of service facilities in each region of two cities, that is, which types of service facilities the region has and which types of service facilities are missing. Furthermore, if the region has this type of service facility, we can count its quantity. For this purpose, we have generated heat maps of the number of different types of service facilities in different regions.

Subsequently, we can calculate the number of disposable service facilities in each community or further calculate the number of disposable service facilities per household. Considering a region separately, then


$$\begin{array}{c} a_{i}=\frac{k}{m}\text{ or }b_{i}=\frac{k}{n} \end{array}$$ (1)

Among them, *a*_*i*_ is the average number of disposable service facilities in the community of the region, *b*_*i*_ is the average number of disposable service facilities per household in the region, k is the total number of facilities of a certain service type in the region, m is the total number of communities in the region, and n is the total number of households in the region.

Finally, considering the convenience level of each service facility, we can use latitude and longitude data to calculate the shortest distance from each community to each service facility. The specific method is to convert latitude and longitude data into numerical types to ensure data consistency and computability. After data preprocessing is completed, the distance is calculated using a correlation function,


$$\begin{array}{c} d_{\min}=\sqrt{({\text{lon}}_{i}-{\text{lon}}_{j}{)}^{2}+({\text{lat}}_{i}-{\text{lat}}_{j}{)}^{2}} \end{array}$$ (2)

Among them, $d_{\min}$ is the shortest distance mentioned above, (lon_*i*_, lat_*i*_) is the longitude coordinate of the community or service facility in the area, and (lon_*j*_, lat_*j*_) represents the latitude coordinate of the community or service facility in the area. The smaller the distance, the more convenient it is for the community to enjoy this type of service, and correspondingly, the service level of this type of service may be higher.

For the subsequent analysis of urban commonalities, a comprehensive quantitative analysis of the service levels of various types can be conducted.

Overall, a comprehensive model has been constructed through density and distance calculations, as well as diverse visualization methods, to evaluate and compare the performance of two cities in terms of facility distribution and accessibility. This not only provides scientific data support for urban planning and resource allocation, but also lays a solid foundation for further analysis and optimization.

### 3.3 An urban resilience model in emergency situations

Afterwards, We have established a diversified urban service system evaluation model to comprehensively and multi dimensionally evaluate the resilience of two cities in the face of extreme weather and emergency situations.

Firstly, we calculate the total area of each region based on relevant latitude and longitude information. The specific method is to convert latitude and longitude data into numerical types to ensure data consistency and computability. After data preprocessing is completed, the area of each administrative division is calculated using correlation functions. This function estimates the total area of each region based on the minimum and maximum latitude and longitude values, and summarizes the total number of households in each region. The calculation of area adopts the rectangular area formula:


$$\begin{array}{c} S_{\text{estimate}}=({\text{lon}}_{\max}-{\text{lon}}_{\min})\times ({\text{lat}}_{\max}-{\text{lat}}_{\min}) \end{array}$$ (3)

In the equation, ${\text{lon}}_{\max}$ (${\text{lon}}_{\min}$) indicates the maximum (minimum) longitude value of the region, ${\text{lat}}_{\max}$ (${\text{lat}}_{\min}$) indicates the maximum (minimum) latitude value of the region. After calculating the total area, we multiplied the calculated area of Changchun and Hohhot by a coefficient between the building area and the total land area. Considering the actual situation, the coefficients for the two cities are 0.2 and 0.03, respectively.

Then calculate the ratio μ of the number n of specific facilities in each area to the area s of that area, that is


$$\begin{array}{c} \mu =\frac{n}{s} \end{array}$$ (4)

Based on the shortest distance we calculated from each community to the nearest facility, we can provide basic supporting data for the subsequent comprehensive evaluation.

We conducted a quantitative analysis and evaluation of the resilience and resilience capabilities of two cities in response to extreme weather and emergency situations using three indicators:

1. Emergency response speed: represents the average distance from each community to the nearest facility, reflecting the emergency response speed of facilities under extreme conditions, that is, the convenience level of the masses;
2. Resource allocation efficiency: represents the density of each facility in a city, reflecting the relative quantity of that facility to a city and providing a macro density evaluation;
3. Per capita facility density: represents the average facility density per household. Unlike resource allocation efficiency, per capita facility density is chosen to be averaged within each community and then averaged across all areas, which better reflects the average level of overall urban facilities in each region and provides a micro level density evaluation.

This evaluation framework essentially constitutes a multi-criteria decision-making problem, requiring the integration of quantitative indicators that cannot be directly compared (such as distance, density, and per capita indicators) with policy requirements such as prioritizing emergency response speed. We employ the Analytic Hierarchy Process (AHP) to formalize the complexity of indicators, which decomposes decisions into a hierarchical structure, obtains expert preferences through pairwise comparisons, and converts them into a weight vector, the internal consistency of which can be verified through statistical methods. In the subsequent ranking phase, we select the Technique for Order Preference by Similarity to an Ideal Solution (TOPSIS), whose geometric principle of measuring the closeness to the ideal optimal solution and the anti-ideal worst solution can produce transparent and easily interpretable results. Urban planners can directly map these results onto budget items. Therefore, the AHP-TOPSIS sequence provides a concise and rigorous approach to transform technical evaluations into persuasive investment strategies.

Following this rationale, we constructed a pairwise comparison matrix to derive the criterion weights. We use the Analytic Hierarchy Process (AHP) to assign weights to each evaluation criterion. For example, when evaluating facility stability, the weight of facility density may be higher, but when evaluating emergency response level, the shortest distance from the community to the facility may be assigned a higher weight.

Subsequently, we constructed a decision matrix using the TOPSIS method to comprehensively evaluate the performance of Changchun and Hohhot under various evaluation criteria. The TOPSIS method will prioritize determining positive ideal solution, A^*^ and negative ideal solution, $A^{\prime }$:


$$\begin{array}{c} A^{*}=(l_{1}^{*},l_{2}^{*},\ldots ,l_{k}^{*}) \end{array}$$ (5)

$$\begin{array}{c} A^{\prime }=(l_{1}^{\prime },l_{2}^{\prime },\ldots ,l_{k}^{\prime }) \end{array}$$ (6)

Among them, $l_{j}^{*}$ is the optimal value of the jth indicator and $l_{j}^{\prime }$ is the worst value of the jth indicator. Then calculate the Euclidean distances between each city and the ideal solution $D_{i}^{*}$ and negative ideal solution $D_{i}^{\prime }$:


$$\begin{array}{c} D_{i}^{*}=\sqrt{\sum_{j=1}^{k}(r_{ij}-l_{j}^{*}{)}^{2}} \end{array}$$ (7)

$$\begin{array}{c} D_{i}^{\prime }=\sqrt{\sum_{j=1}^{k}(r_{ij}-l_{j}^{\prime }{)}^{2}} \end{array}$$ (8)

Among them, *r*_*ij*_ is the corresponding indicator for the city. By measuring the distance between each city and the ideal and negative ideal solutions, a comprehensive score is obtained to rank the cities based on their strengths and weaknesses. In order to further refine the analysis, we set evaluation criteria and estimated the scores of various facilities in each city under three key indicators, and displayed the distribution of these indicators through visual means.

### 3.4 Long-short term investment planning model

Finally, based on the TOPSIS scoring results, we propose targeted development recommendations for the two cities to guide the optimization direction of infrastructure construction and resource allocation, and assist in the resilience of the cities.

In the funding allocation issue in the fourth development report, we have designed the following model.

In the budget allocation process, the weights of three key indicators were first determined based on the AHP method, where the proportion of emergency response speed is $\sigma_{1}=52.8\%$. The efficiency of resource allocation is $\sigma_{2}=33.3\%$, the per capita facility density is $\sigma_{3}=14\%$. For each facility, when calculating the short-term gap (we take the gap as 100 minus the normalized score), multiply the gaps in emergency response speed and resource allocation efficiency by their corresponding weights, and then add them up to obtain the total short-term gap, $\tau_{1}$:


$$\begin{array}{c} \tau_{1}=\sigma_{1}\alpha_{1}+\sigma_{2}\alpha_{2} \end{array}$$ (9)

Among them, $\tau_{1}$ represent the short-term total gap, $\alpha_{1},\alpha_{2}$ represent the weights of emergency response speed and resource allocation efficiency, and $\alpha_{1},\alpha_{2}$ represent the gaps in emergency response speed and resource allocation efficiency. The long-term gap is the gap in per capita facility density multiplied by its weight,


$$\begin{array}{c} \tau_{2}=\sigma_{3}\alpha_{3} \end{array}$$ (10)

Among them, $\tau_{2}$ is the long-term total gap, $\sigma_{3}$ is the weight of per capita facility density, $\alpha_{3}$ and is the per capita facility density gap.

Next, sum up the short-term and long-term gaps of all facilities separately to obtain the overall total short-term and long-term gaps. The proportion of each facility in short-term and long-term investment is the ratio of its shortfall to the total shortfall. If the total gap is zero, the corresponding investment ratio is set to zero to avoid the error of zero.

Finally, allocate the total budget in a ratio of short-term to long-term (60% for short-term and 40% for long-term), and calculate the specific short-term and long-term investment amounts based on the investment ratio of each facility.

This method ensures that the budget is allocated reasonably based on the degree of deficiency and importance of each facility in key indicators, thereby effectively enhancing the robustness and resilience capacity of urban infrastructure.

## 4 Results

### 4.1 Quantitative analysis of urban service level

**Fig 5:**
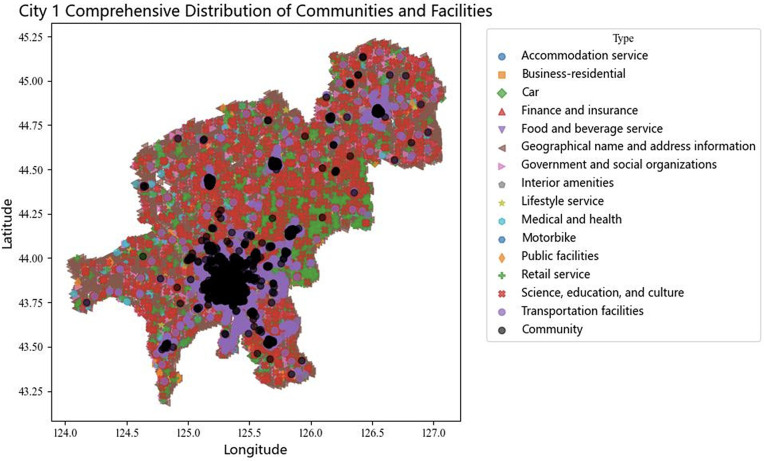
Distribution of all communities and 15 types of service facilities in Changchun.

**Fig 6:**
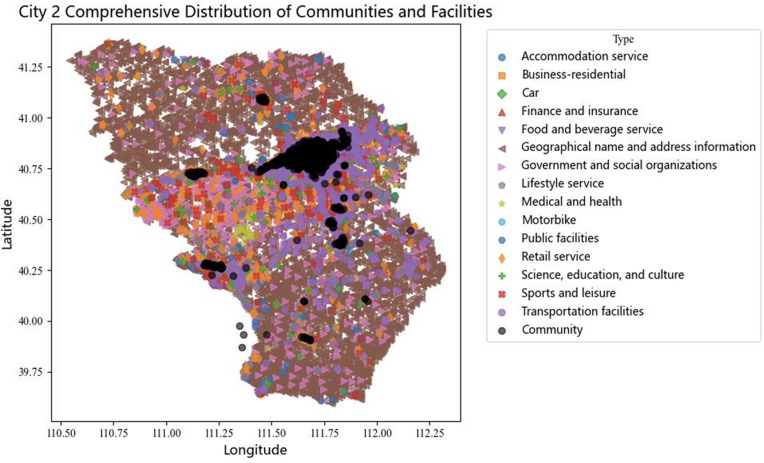
Distribution of all communities and 15 types of service facilities in Hohhot.

Firstly, based on the coordinates of the two urban communities and the coordinates of different types of service facilities, we have created distribution maps of all communities in the two cities and the 15 types of service facilities, as shown in Figs 5 and 6. We have marked the corresponding locations of all communities in black. Upon initial observation, it is not difficult to see that the distribution of various types of service facilities is relatively concentrated in areas where the black dots (communities) are more concentrated. Among them, the distribution of transportation facilities is particularly dense around the community, which precisely facilitates the travel needs of urban residents, ensures the mobility of urban transportation, and meets the planning needs of the city.

**Fig 7:**
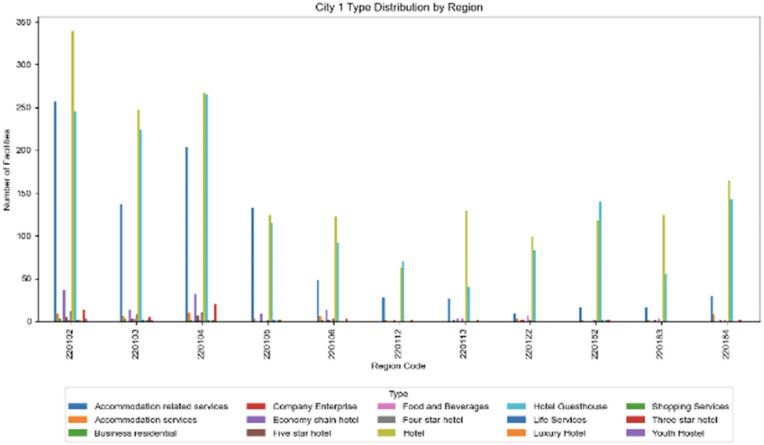
Bar chart of the distribution quantity of different types of service facilities in different regions for Changchun (left)/Hohhot (right).

At the same time, we have drawn multiple comparative images of service systems in Changchun and Hohhot, as shown in Fig 7, which is a bar chart of the distribution of different types of service facilities in different areas of the two cities. After understanding the approximate distribution of communities and service facilities, we quantitatively calculated the indicators in the model constructed before.

**Fig 8:**
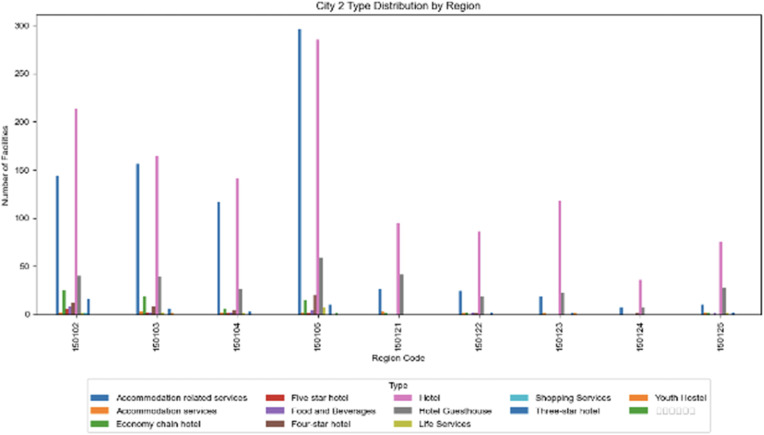
Heat map of the available quantity of different types of service facilities in communities in different regions for Changchun (left)/Hohhot (right).

We have also drawn a heatmap of the average disposable quantity of different types of service facilities in each community in different regions, as shown in Fig 8. In this figure, we have standardized the data to provide a certain reference standard for each value, which makes the comparison more convincing.

Through preliminary observation, we can understand the richness of service facility types in each region. If the region has more service facilities of that type, the corresponding color in the graph will be closer to red; On the contrary, if the area lacks a certain type of service facility, the corresponding color in the figure will be closer to blue. Specifically, if the area does not have that type of service facility, the corresponding color in the figure will be blue and the corresponding number will be 0. Thus, we can learn about the richness of service facilities in different regions.

Secondly, we found that different types of service donations are distributed in different regions of each city, and some basic services closely related to daily life are densely distributed. The complete service facilities provide local residents with a good living experience and can also help solve various living problems.

Furthermore, we calculated the average shortest distance from each community in two cities to various types of service facilities (standardized here), as shown in Table 1. The smaller the distance, the more convenient it is for community residents to enjoy this service. Observing the table below, we found that the distance between the community and various types of service facilities is relatively small, and the public can easily enjoy various service facilities.

**Table 1:** Average Shortest Distance between Communities and Various Types of Service Facilities in Two Cities.

Nearest Facility		Average Distance
Changchun	Accommodation service	0.00358
	Business-residential	0.00007
	Finance and insurance	0.00347
	Car	0.00252
	Food and beverage service	0.00129
	Geographical name and address information	0.00076
	Government and social organizations	0.00096
	Interior amenities	0.10676
	Medical and health	0.00169
	Lifestyle service	0.00104
	Motorbike	0.00011
	Public facilities	0.00521
	Retail service	0.00058
	Science, education, and culture	0.00154
	Transportation facilities	0.00071
Hohhot	Accommodation service	0.00385
	Business-residential	0.00138
	Car	0.00257
	Finance and insurance	0.00319
	Food and beverage service	0.00124
	Geographical name and address information	0.00091
	Government and social organizations	0.00170
	Lifestyle service	0.00098
	Medical and health	0.00173
	Motorbike	0.00246
	Public facilities	0.00241
	Retail service	0.00097
	Science, education and culture	0.00164
	Sports and leisure	0.00271
	Transportation facilities	0.00770

It can be seen that both cities have a high level of service, which is not only reflected in the richness of service facilities, but also in the convenience of service facilities. Specifically, the numerical values of the heat map can to some extent reflect the service level of different types of facilities. For example, in Changchun, the service level of food and beverage service, lifestyle service, etc., is higher, while the service level of motorbikes is lower.

We can also summarize the commonalities between the two cities from the chart. Firstly, there are regional differences in the distribution of service facilities between the two cities, that is, there are certain differences in the density of service facility distribution in different regions. For example, in Changchun, the distribution of various service facilities is concentrated in the areas with address code 220105 and address code 150121 in Hohhot, while in Changchun, the distribution of various service facilities is sparse in the areas with address code 220112 and address code 150122 in Hohhot. This may be related to the high or low living needs of the local people. Secondly, the retail services in the two cities are most densely distributed, with the highest density even reaching 1458.78. As the service facility most closely connected to daily life, this service undoubtedly provides the greatest living support for the community and meets people’s supply and demand. Finally, both cities have one or several areas where each type of service facility is densely distributed. For example, in Changchun with an address code of 220105 and in Hohhot with an address code of 150121, we speculate that these areas may be the most economically developed regions of both cities.

However, there are also certain differences between the two cities. For example, the transportation system of Changchun is obviously more complete than that of Hohhot, and correspondingly, the indicators of Car and Motorbike in Changchun are relatively higher than those in Hohhot. For example, Changchun lacks sports and leisure indicators, while Hohhot lacks interior amenities indicators.

In summary, Changchun has more developed transportation and denser service systems, but overly dense service systems may also lead to problems such as urban overload, environmental damage, and high resource consumption; Hohhot may be more conducive to urban governance, environmental protection, and personalized services, but it may face problems such as insufficient service facilities and lack of service resources.

### 4.2 Results of the urban resilience model in emergency situations and Long-Short Term Investment Planning Model

We conducted a quantitative analysis and evaluation of the resilience and resilience capabilities of two cities in response to extreme weather and emergency situations using three indicators:

1. Emergency response speed: represents the average distance from each community to the nearest facility.
2. Resource allocation efficiency: represents the density of each facility in each region.
3. Per capita facility density: represents the facility density shared by each household.

Ultimately, we use infrastructure robustness to represent the service level of urban infrastructure construction and the comprehensive stability in responding to extreme natural disasters. As the emergency response speed of a city is crucial for the stability of urban development when natural disasters occur, we agree that infrastructure robustness is the weighted sum of emergency response speed, resource allocation efficiency, and per capita facility density in AHP.

Based on the previous modeling results, we obtained the following results:

Firstly, for the Analytic Hierarchy Process (AHP), we obtained the importance weights of three indicators through solving, as shown in the Table 2.

**Table 2:** Weight Table of Various Service Level Evaluation Indicators.

Emergency Response Speed	Resource Allocation Efficiency	Per Capita Facility Density
0.528	0.333	0.14

**Fig 9:**
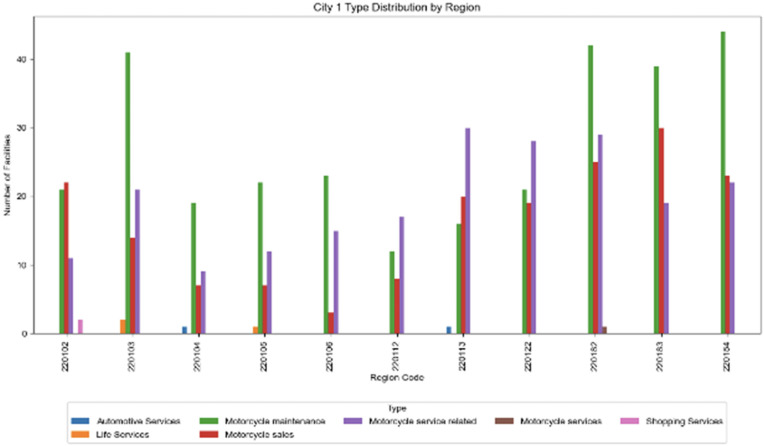
Evaluation Chart of Resilience Indicators for Changchun (Left)/Hohhot (Right).

**Fig 10:**
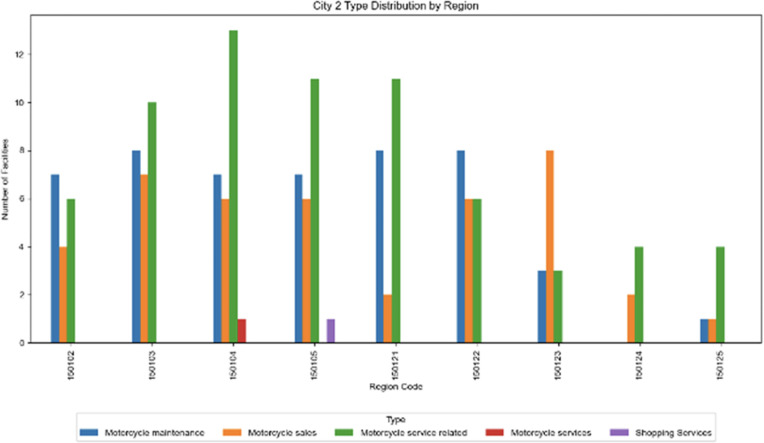
Future Investment Plan for Changchun (Left)/Hohhot (Right).

After conducting relevant analysis using TOPSIS, we drew resilience index evaluation charts for each city as shown in Fig 9, as well as service level scores for various service facilities as shown in Table 3 and we listed the Long-term and short-term investment plans as shown in Table 4 and Fig 10. We found that for Changchun, due to its large population and area, in addition to geographical villages and mountains, the robustness scores of retail and living service infrastructure are relatively high, reaching 82.81970237 and 81.02532504 points respectively, while public facilities and facilities related to finance and insurance industries have lower scores. In terms of individual indicators, the retail services and commercial residential areas in Changchun have performed well in emergency response speed, demonstrating their ability to fully respond to sudden disasters. However, the motorcycle services and public facilities related fields have performed poorly, making it difficult to make quick decisions in the event of a sudden crisis. In terms of resource allocation efficiency, motorcycle services and living service facilities have strong guarantee capabilities, but financial, insurance, and accommodation related services cannot meet allocation needs. For residents in Changchun, per capita facility density can better reflect a city’s level of resource allocation. Under this indicator evaluation standard, retail and catering services can better meet urban demand.

**Table 3:** Rating of Service Levels for Various Service Facilities in Changchun and Hohhot.

Facility		Emergency	Resource	Per Capita	Total
Changchun	Accommodation services	60.40725007	58.12618310	53.01209515	58.67284414
	Business-residential	89.53476098	67.18310221	55.22070782	76.929823
	Car	61.97141704	64.08792824	56.69928344	62.4211371
	Finance and insurance	60.17981499	56.97961476	53.06202113	58.483777
	Food and beverage service	79.54373111	72.16858262	88.53561408	77.9848651
	Geographical name and address information	100	68.21626989	88.53567147	87.9110118
	Government and social organizations	68.785755	64.31872121	58.13306171	65.81991766
	Interior amenities	50	76.31649238	56.46242606	59.7181316
	Lifestyle service	82.1266829	72.70835525	78.51567061	81.0253250
	Medical and health	72.86941738	72.21961411	60.68167013	70.823363
	Motorbikes	53.86694574	100	50	68.745311
	Public facilities	57.03522013	50	51.12754697	53.9224528
	Retail service	90.69871502	65.80655224	100	82.8197024
	Science, education, and cultures	65.28715022	65.26754908	61.89305625	70.1513431
Hohhot	Accommodation services	63.33065045	99.90149969	52.898805	74.1116049
	Business-residential	81.988152	80.23113203	55.71153301	77.6741837
	Car	65.21159269	50	54.54116301	58.7714830
	Finance and insurance	61.48866467	97.35905606	52.64994982	72.2575630
	Food and beverage service	85.82693744	67.35063668	56.69928344	78.80633352
	Geographical name and address information	100	73.5198845	64.64875714	86.98633352
	Government and social organizations	61.98866467	60.3198845	56.46242606	66.7419876
	Lifestyle service	96.3621219	79.48248506	75.5699506	87.9266644
	Medical and health	76.01777617	77.29826238	57.86292533	73.9750607
	Motorbike	50	53.97916128	50	51.7850167
	Public facilities	66.47209555	100	52.5361423	75.7525324
	Retail service	96.5892798	60.02760855	100	86.98633352
	Science, education and culture	76.06896699	54.60027608	57.86292533	68.67284414
	Sports and leisure	64.24790617	70.37302173	53.53298671	64.7760898

**Table 4:** Long- and Short Term Investment Planning for Changchun and Hohhot.

City		Short Term Investment	Long Term Investment
Changchun	Accommodation service data	5454823806	3543599296
	Business-residential data	2668236691	3339225949
	Car data	4752795859	3341574580
	Finance and insurance data	5466240692	3538813470
Hohhot	Food and beverage service data	3199084515	11029827636
	Accommodation service data	4157129081	11110628415
	Business-residential data	1804723074	18597387717
	Car data	3895014803	19088843417
	Finance and insurance data	2359528392	19882992151
	Food and beverage service data	2045177471	901687066.5

In summary, for Changchun, public facilities and the financial and insurance industries have become key areas for its short-term future development in the comprehensive evaluation system, and are more likely to become its shortcomings and weaknesses in the face of sudden natural disasters. Based on this, we checked the annual financial expenditure of Changchun through the Internet and formulated the following possible short-term and long-term investment plans for Changchun, assuming that a total of 100 billion yuan will be invested and spent. Due to the urgent need to strengthen public facilities and the financial insurance industry, the model suggests that a large amount of funds need to be invested in these areas in the short term to ensure the normal development of the economy and daily consumption of residents. For commercial residential buildings and living service facilities with high scores in the current evaluation model, less funding can be invested in the short term. In the long run, efforts should be made to increase the protection of villages and nature, in line with current ecological strategies and resilience plans.

For Hohhot, the efficiency of resource allocation is slightly lower than that of Changchun. The number and density of “motorcycles” and “vehicles” facilities in Hohhot cannot meet the needs of residents. At the same time, the scientific, educational, and cultural resources in Hohhot are relatively scarce, and the resource allocation efficiency and per capita facility density of “scientific, educational, and cultural” facilities are low. For Hohhot, due to its small population and area, the robustness scores of the related infrastructure, including living service facilities and retail service infrastructure, are relatively high, reaching 87.9266644 and 86.98633352 points respectively. However, the scores of the industry facilities related to automobile and motorcycle services are relatively low. In terms of various individual indicators, the retail and catering services in Hohhot have performed well in emergency response speed, demonstrating their ability to fully respond to sudden disasters. However, the accommodation services and financial insurance related industries have performed poorly, unable to make quick decisions effectively when sudden crises arise. In terms of resource allocation efficiency, public facilities and accommodation facilities have strong guarantee capabilities, but related services such as automobiles and science, education, and culture cannot meet allocation needs. For residents in Hohhot, per capita facility density better reflects a city’s level of resource averaging. Under this indicator, retail and catering services can better meet urban demand, but motorcycle and public facility services need to be improved.

In summary, for Hohhot, automobile services and motorcycle industry have become key areas for its short-term future development in the comprehensive evaluation system, and are more likely to become its shortcomings and weaknesses in the face of sudden natural disasters. Based on this, we checked the annual financial expenditure of Hohhot through the Internet and formulated the following possible short-term and long-term investment plans for Hohhot, assuming that a total of 60 billion yuan will be invested and spent.

Due to the urgent need to strengthen the motorcycle and automobile service industries, the model suggests that more funds need to be invested in public facilities and financial insurance industries in the short term to ensure the normal development of the economy and daily consumption of residents. For commercial residential buildings and living service facilities with high scores in the current evaluation model, less funding can be invested in the short term. In the long term, it is necessary to increase investment in related services and public facilities in the motorcycle industry to promote sustainable economic development. At the same time, it is still necessary to maintain investment in other facilities, strengthen infrastructure construction, ensure long-term stable living for residents, and enhance the ability of cities to prevent and respond to sudden natural disasters.

## 5 Discussion

### 5.1 AHP consistency and sensitivity analysis

**Fig 11:**
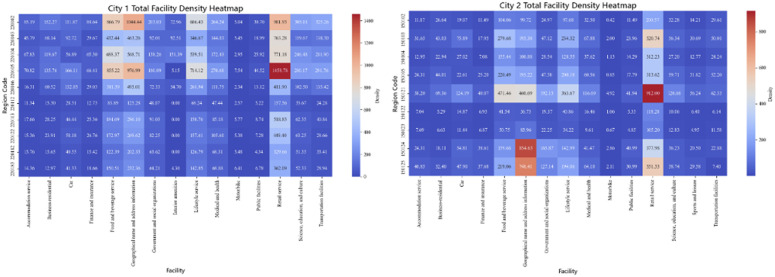
Results of AHP Consistency Analysis.

**Fig 12:**
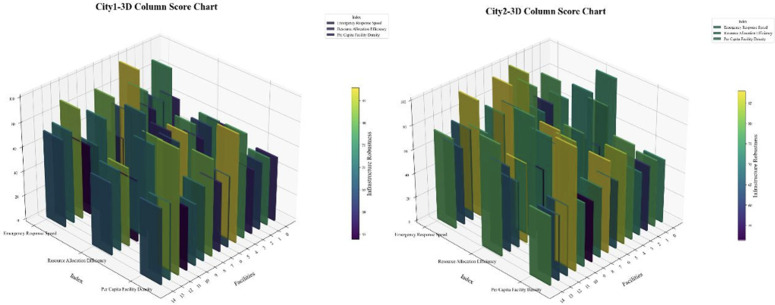
AHP Sensitivity Analysis for Changchun (a) / Hohhot (b).

Following the consistency and sensitivity experiments, the following conclusions have been drawn. Fig 11 illustrates the results of the AHP (Analytic Hierarchy Process) consistency analysis, while Fig 12 presents the sensitivity test outcomes of the AHF (Analytic Hierarchy Function, presumably a variation or specific application of AHP methodology) for two cities. The consistency ratio of the Analytic Hierarchy Process (AHP) is 0.04622549644738945, which is significantly lower than the criterion of 0.1, indicating that the pairwise comparisons made by experts or decision-makers possess a high degree of consistency. This implies that the determined weights of various indicators are reasonable and can effectively reflect the significance of each evaluation criterion in practical applications. Consequently, these weights can be confidently employed in subsequent comprehensive evaluation calculations.

From the sensitivity analysis results, it is observed that for both Changchun and Hohhot, the overall evaluation scores exhibit varying degrees of fluctuation in response to changes in the weights of different indicators. For Changchun, the “Emergency Response Speed” indicator significantly influences the overall score, increasing by approximately 0.1424 when the weight is positively perturbed by 10%, and decreasing by about 0.3187 when the weight is reduced by 10%. This demonstrates the indicator’s high sensitivity. In terms of the “Resource Allocation Efficiency” indicator, a 10% decrease in weight leads to a substantial drop in the overall score (approximately 0.22385), whereas an increase in weight results in a minor rise (only about 0.08208). The “Per Capita Facility Density” indicator impacts Changchun asymmetrically; a reduction in weight causes the overall score to decline by about 0.15203, whereas an increase in weight results in an almost negligible change (only about 0.00224). This asymmetric effect suggests that slight adjustments in the weights of different indicators during the decision-making process may lead to complex and uneven impacts on the final evaluation outcomes.

In contrast, the sensitivity analysis for Hohhot reveals that the “Emergency Response Speed” indicator has a minimal impact on the overall score with a 10% weight perturbation, increasing by only about 0.01037 with a positive change and decreasing by about 0.17565 with a negative perturbation. The “Resource Allocation Efficiency” indicator exhibits a similar symmetric effect, with a positive increase of approximately 0.01100 and a negative decrease of about 0.17258. However, the “Per Capita Facility Density” indicator significantly affects Hohhot, with a 10% decrease in weight leading to a substantial drop in the overall score (about 0.25964), and an increase in weight causing an increase of about 0.10933. This indicates that for Hohhot, the “Per Capita Facility Density” indicator is highly sensitive, and changes in its weight can significantly alter the overall evaluation results.

Overall, these sensitivity results illustrate that different cities vary in the extent to which various indicators influence them, particularly the “Emergency Response Speed” and “Per Capita Facility Density” indicators, which exhibit strong volatility under different scenarios. In practical decision-making, it is necessary to conduct an in-depth analysis of these key indicators in conjunction with actual conditions and consider a range of weight settings to ensure that the comprehensive evaluation results possess sufficient robustness and representativeness.

### 5.2 Shortcomings and prospects

In the modern urban resilience, we can intuitively evaluate the level of urban resilience through the indices generated by the model, taking into account the city’s ability to face extreme weather and emergency situations; It can also integrate various types of services in the city from multiple aspects, thus providing resilience strategies that are more in line with the actual situation of the city and promoting high-quality urban development.

However, in the process of calculating the number of disposable facilities (facility density) in each community, we divide the total number of facilities in the area by the number of communities, but ignore the individual differences of each community, and the different situations of each community may result in the calculated mean only having a certain reference value, without actual evaluation criteria. For example, although there are the same number of service facilities S near community A and community B, and the distance between the two to the service facility is comparable, the total number of households in community A is much larger than that in community B. Therefore, the competitiveness of residents in community A for the service facility is much greater than that of residents in community B. As a result, community A enjoys a much lower level of service from service facility S than community B. However, through the average value, we can only know that community A and community B can simultaneously obtain the same service facility S, but cannot analyze the service results concretely. More specifically, even if Community A and Community B have similar situations (including roughly the same number of households and residents’ travel levels), we still overlook the different service quality issues of different service facilities. For example, the medical level of national tertiary hospitals is much higher than that of community hospitals. Therefore, our service level evaluation indicators can only objectively consider the completeness of service facilities and the degree of dispersion and density of facility construction in the city, and there may be some errors in the overall service level analysis results.

### 5.3 Future decision-making or resource management in Urban planning

While the preceding sections demonstrate that our AHP-TOPSIS framework can quantify the relative weaknesses of each urban district, the ultimate value of any assessment model lies in its ability to guide concrete administrative action. To bridge the gap between “evaluation” and “execution”, we translate the calculated short-term and long-term investment ratios ($\tau_{1}$ and $\tau_{2}$) into three immediately usable policy instruments: fiscal programming, land-use regulation and infrastructure procurement.

First, at the fiscal level, the $\tau_{1},\tau_{2}$ values in Table 4 can be inserted directly into the municipal capital-budget circular. Taking Changchun as an example, the 2025−2027 urban infrastructure special-budget envelope is pre-set at RMB 100 billion; districts whose $\tau_{1}$ share exceeds the city mean by more than one standard deviation (i.e., public-facilities and finance-insurance rows) are automatically elevated to “Tier-1 priority”, allowing them to draw on the 60% front-loaded portion of the envelope. Conversely, districts with $\tau_{2}$ below the mean (retail and food-beverage services) will receive only maintenance funds for the first two years, thereby hard-wiring the model output into the annual budgeting timetable without additional political negotiation.

Second, at the land-use level, the per-capita facility density score (one of the three AHP-weighted inputs) is written into the forthcoming Changchun and Hohhot statutory detailed-planning regulations. Any development parcel whose 500 m service-circle density falls below 14% of the city-wide average must now provide on-site emergency redundancy of no less than 3% of its total gross floor area, dedicated to community shelters, distributed-energy rooms or micro – fire stations. This “density-offset” clause converts a previously abstract resilience metric into a legally enforceable planning condition, ensuring that new growth finances part of the resilience deficit rather than simply exporting it to older neighborhoods.

**Fig 13:**
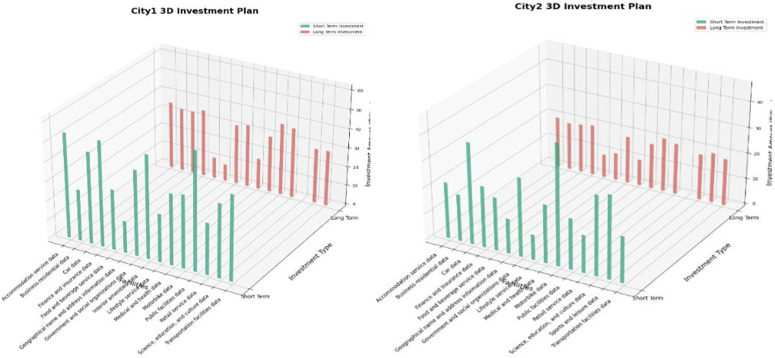
Future investment plan for Changchun (Left) and Hohhot (Right). Three-dimensional bar charts visualizing the short-term (green) and long-term (red) investment allocations across 15 categories of urban service facilities. The vertical axis represents investment amounts in hundred million yuan. Short-term investments account for 60% of the total budget, while long-term investments account for 40%, allocated proportionally based on the TOPSIS-derived gap ratios (τ ₁ and τ ₂) for each facility category.

**Fig 14:**
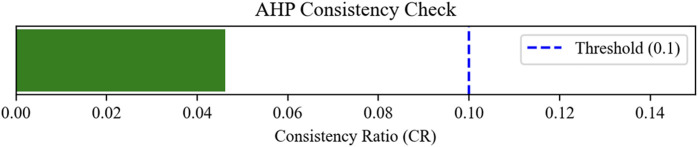
Results of AHP consistency analysis. The consistency ratio (CR) of the Analytic Hierarchy Process is 0.046, which falls well below the acceptable threshold of 0.1 (dashed blue line). This indicates a high degree of consistency in the expert pairwise comparisons, confirming that the derived criterion weights are reliable for subsequent comprehensive evaluation calculations.

**Fig 15:**
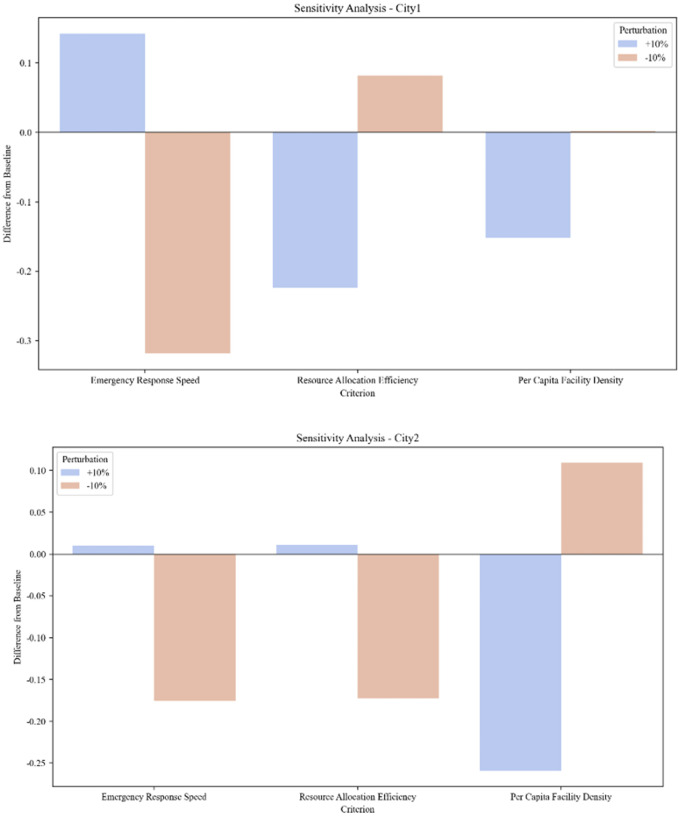
AHP sensitivity analysis for Changchun (a) and Hohhot (b). Bar charts showing the deviation of overall resilience scores from baseline when the weights of the three evaluation criteria—Emergency Response Speed, Resource Allocation Efficiency, and Per Capita Facility Density—are perturbed by +10% (blue) and −10% (orange). Positive deviations indicate score increases, while negative deviations indicate score decreases relative to the baseline.

Third, at the procurement level, the short-term investment amounts in Table 4 are packaged into two-year performance-based contracts. For Hohhot, the model allocates RMB 3.9 billion to “car and motorcycle services” because $\tau_{1}$ for that category ranks highest; the municipal transport bureau therefore issued an invitation-to-tender in 2024-Q2 that explicitly scores bidders on (i) average shortest distance to community centroids (<0.002°) and (ii) POI-normalised density uplift (> 15%), mirroring the two highest-weighted indicators in our AHP layer. Thus, contractor selection is driven by the same arithmetic that produced the resilience rankings, guaranteeing consistency between technical evaluation and on-the-ground delivery (Figs 13–15).

Finally, to ensure that these instruments remain adaptive, we recommend embedding the AHP weight-set as a scheduled review item every three years, coinciding with the statutory revision of comprehensive urban plans. If extreme-weather event logs or post-event after-action reviews reveal a shift in criticality (e.g., consecutive heat-waves elevate medical-facility distance above the current 52.8% weight threshold), the pairwise comparison matrix can be re-solved and the new $\tau_{1},\tau_{2}$ shares propagated through budget, land and procurement codes in the same manner. By explicitly reserving administrative entry points for model outputs, the discussion moves beyond “our city should be more resilient” to next fiscal year, District X will receive Y billion, and every new parcel must build Z m^2^ of emergency space, because the numbers say so.

## 6 Conclusion and outlook

The current situation of declining population, increasingly frequent global extreme weather events, and economic recession will pose unprecedented challenges to the resilience of urban development, inevitably affecting the high quality and resilience of manycities. In the face of the current demand of domestic cities to improve urban resilience and enhance resilience, we have established an urban resilience assessment system in the event of sudden natural disasters based on housing data, infrastructure data in 25 regions of two cities, and GDP and population data in each region of cities obtained from the Internet. In the emergency assessment system for urban resilience in the event of sudden natural disasters, we considered and analyzed various infrastructure service levels of two cities from multiple dimensions, and designed a model based on this. In the urban emergency resilience evaluation system, we have introduced three evaluation indicators: emergency response speed, resource allocation efficiency, and per capita facility density. We use Analytic Hierarchy Process (AHP) to analyze importance weights and obtain robustness of comprehensive infrastructure. Based on this, we used TOPSIS method to analyze the advantages and disadvantages of the current service levels of two cities, extracted their common and unique features as well as specific weaknesses, and discussed the main investment areas and amounts of the two cities under limited financial resources constraints. We also provided long-term and short-term investment strategies and reports for smart city construction, and predicted the expected improvement of the city’s smart development level. Finally, we conducted an error analysis and discussed the advantages and disadvantages of the model, and found that existing models can to some extent reflect the stability and resilience level of cities. This model is of great significance in quantitatively analyzing urban resilience and resilience capabilities, and can provide important reference opinions for cities to make decisions on resilience improvement and resilience in the future, which will help promote the construction of smart cities and urban resilience in emergency situations.

## Supporting information

S1 File Dataset. (ZIP)
