# Supplementary material for: Design of Urban resilience model in emergency situations
Source: PLoS One. 2026 Jun 9;21(6):e0347588. doi: 10.1371/journal.pone.0347588 (PMC13249160; doi:10.1371/journal.pone.0347588)
Supplement: Attachment — Submitted filename: Response to Reviewers.docx [file pone.0347588.s003.docx]

Answer to Reviewer

1. Please clarify the full term of the abbreviation “GDP” in the abstract.

Thank you for pointing this out. The abbreviation “GDP” stands for Gross Domestic Product. It is a key economic indicator that measures the total value of goods and services produced over a specific time period. In our study, GDP data were used as one of the factors to assess the economic resilience of cities, alongside other indicators such as population density and infrastructure levels. We have now explicitly defined “GDP” in the abstract to avoid any confusion.

2. In the introduction, when discussing prior studies, please provide more detailed information regarding their evaluation methods, datasets, and the strengths and limitations of their approaches. This will help situate your work more clearly within the existing literature.

We appreciate your suggestion to provide more detailed information on prior studies. We have revised the introduction to include more comprehensive descriptions of the evaluation methods, datasets, and strengths and limitations of previous research. For example, we have expanded our discussion on the work of Beheshtian et al. (2018), who used climatic data and energy consumption metrics to assess transportation resilience in New York City. Their study highlighted the importance of integrating multi-sector data but faced limitations in terms of spatial resolution and real-time data integration. Similarly, we have added more details on other studies, such as those by Elmqvist et al. (2019) and Meerow et al. (2016), to provide a clearer context for our work within the existing literature.

3. Have any previous studies assessed urban resilience using POI (Point of Interest) data? If such work exists, it should be cited and compared with your approach.

Thank you for raising this important point. Indeed, there have been several studies that have utilized Point of Interest (POI) data to assess urban resilience. For instance, Chen et al. (2024) used social media data linked to POI information to evaluate urban vibrancy during the COVID-19 pandemic. Another example is the work by Zhong et al. (2025), who integrated POI data with geospatial analysis to assess green space accessibility in the Yangtze River Delta. We have now cited these studies and compared them with our approach, highlighting how our work builds on previous efforts while introducing new dimensions such as the integration of economic parameters and comprehensive investment planning.

4. The manuscript gives limited attention to related work and currently lacks a dedicated literature review section. Including a structured and comprehensive related-work section would significantly strengthen the theoretical grounding of the study.

We agree that a dedicated literature review section would significantly enhance the theoretical grounding of our study. We have therefore added a new section titled “Related Work,” where we systematically review the existing literature on urban resilience. This section provides an overview of key concepts, methodologies, and findings from previous studies. It also discusses the gaps in current research and how our study addresses these gaps. We believe this addition will provide readers with a more comprehensive understanding of the context and significance of our work.

5. Please elaborate on the key components and parameters traditionally used to measure urban resilience in the literature. How do the indicators you selected—such as parking fees or housing prices—fit within the established conceptual frameworks of urban resilience?

Existing studies typically treat POIs as simple count indicators for single hazards or single functions, assign weights subjectively to produce heat maps, lack coupling with economic parameters, and do not answer the question “with a limited budget, where should investment go first?” In contrast, this study incorporates fifteen POI categories into a three‑dimensional “distance–density–economy” matrix, derives emergency‑scenario weights via AHP, and uses TOPSIS to convert each district’s distance to the ideal solution into an investment‑gap ratio. Short‑ and long‑term investments are then allocated proportionally, yielding a concrete roster of facilities and investment amounts (in units of 100 million yuan), thus filling the gap between having indicators and having an implementable plan.

Traditional frameworks emphasize five pillars—social, economic, environmental, physical, and institutional—but often stall in practice because indicators are difficult to quantify, weights are hard to reach consensus on, and results are hard to translate into actionable investments. In our framework, for example, housing prices and parking fees are modeled as dual “economic–physical” factors: housing price deviation captures affordability, while parking price differentials reflect transportation structure. These two factors jointly adjust per‑capita facility density scores, allowing price signals and spatial signals to interact within the same vector. After AHP–TOPSIS integration, districts with high housing prices and low facility density naturally shift toward the negative ideal and receive higher investment priority, thereby converting the abstract objectives of the five pillars into expressions that are actionable, budgetable, and trackable.

6. There are numerous methodological options for problems similar to yours. Please justify why AHP and TOPSIS were specifically chosen and explain why alternative methods were not considered or discussed.

We appreciate the reviewer’s request for clarification. The problem addressed in our study is a multi‑criteria decision problem that combines qualitative expert judgments with quantitative indicators; therefore we selected methods that together (1) elicit reliable criterion weights from expert judgments and (2) produce an interpretable ranking of alternatives.

AHP was chosen because it supports hierarchical structuring of complex criteria, converts pairwise subjective judgments into numerical weights, and provides a consistency ratio to check the reliability of those judgments. This makes AHP particularly suitable when expert preferences are important and need formal consistency testing.

TOPSIS was selected because it ranks alternatives by their distance to an ideal and anti‑ideal solution, which is intuitive, computationally efficient, and easily interpretable by practitioners and stakeholders. TOPSIS integrates naturally with externally derived weights (e.g., from AHP) and yields transparent ranking results.

The combined AHP+TOPSIS workflow leverages complementary strengths: AHP provides defensible, consistency‑checked weights; TOPSIS delivers a clear, reproducible ranking based on those weights. This combination is widely used in applied engineering, management, and policy studies for its balance of rigor and interpretability.

We did consider alternative MCDM methods (e.g., entropy weighting, VIKOR, PROMETHEE, MAUT, fuzzy AHP). These were not adopted for the following practical reasons: entropy weighting relies solely on objective data and does not capture expert preference; VIKOR focuses on compromise solutions which were not the objective here; PROMETHEE requires specifying preference functions that reduce transparency for our audience; MAUT demands detailed utility elicitation and extensive data; fuzzy approaches increase modelling complexity and reduce reproducibility given our data and time constraints. Thus the alternatives were excluded based on suitability, data requirements, complexity, and interpretability considerations.

The selection of AHP and TOPSIS was not driven by “methodological preference,” but rather a rational choice constrained by data attributes, decision objectives, and budgetary context. This paper addresses a typical multi-criteria, low-sample, high-stakes public investment decision: “Should cities invest in infrastructure during sudden extreme weather events, and where should they prioritize?” With 25 districts/counties, 15 facility types, and 3 major indicator dimensions, the sample size is far too small to support the training-validation split required for machine learning. Additionally, the indicators feature mixed units (distance-density-property prices), and the final output must be an interpretable ranking of “who gets priority and how much funding,” not merely predicted probabilities. AHP's value in this scenario lies in explicitly structuring “expert judgment”: it translates the difficult-to-quantify policy preference of “preserving lives and maintaining operations during emergencies” into a testable weight vector through pairwise comparisons. The consistency ratio of 0.046 < 0.1 also demonstrates logical coherence in decision-making. More importantly, it allows the subjective amplification of “emergency response speed” to 52.8%, aligning with the disaster management consensus that “time is paramount”—a normative value that purely data-driven methods cannot a priori incorporate. TOPSIS then seamlessly integrates the weights generated by AHP, quantifying the ideal utopia (“shortest distance-highest density-most affordable housing”) and the worst nightmare. By calculating Euclidean distances, it determines each district's relative position to the “ideal solution,” naturally producing a rankable proximity index. This index can be linearly mapped to fiscal gaps, directly translating into specific investment allocations—60% for short-term and 40% for long-term—enabling seamless integration between ranking and budgeting. In contrast, while entropy weighting or principal component analysis are objective, they excessively amplify high-variance indicators like “housing price differences,” drowning out critical low-variance factors like ‘distance’; DEA excels at evaluating “efficiency frontiers” but fails to handle the cross-impact of price signals and spatial signals; structural equation or factor analysis requires large-sample survey data, whereas this study only has aggregated district/county indicators; Machine learning methods (Random Forest, XGBoost) can capture nonlinearity, but with small samples and few variables, they are prone to overfitting. Moreover, their “black box” outputs make it difficult to justify to finance and audit departments why District A receives 3 billion yuan while District B gets only 500 million. Therefore, the authors explicitly state in the methodology section: Given limited data scale, mandatory decision transparency, and the requirement for one-to-one correspondence between weights and fiscal items, the AHP-TOPSIS combination offers the shortest path to an “explainable, verifiable, and implementable” solution. Consequently, other data-driven or black-box methods were excluded from consideration.

7. The practical implications of your findings should be articulated more clearly. For instance, how can the results contribute to improving future decision-making or resource management in urban planning? These points should be explicitly addressed in the conclusion.

We have revised the discussion to more explicitly address the practical implications of our findings. Our study provides actionable insights for urban planners and policymakers by identifying key areas for investment and improvement in urban resilience. For example, our results highlight the need for enhanced public facilities and financial services in Changchun, and improved transportation infrastructure in Hohhot. We have also discussed how our findings can inform future decision-making in urban planning, such as through fiscal programming, land-use regulation, and infrastructure procurement. By translating our model outputs into specific policy instruments, we aim to bridge the gap between evaluation and execution, ensuring that our research directly contributes to practical urban planning efforts.
