# Supplementary material for: Design of Urban resilience model in emergency situations
Source: PLoS One. 2026 Jun 9;21(6):e0347588. doi: 10.1371/journal.pone.0347588 (PMC13249160; doi:10.1371/journal.pone.0347588)
Supplement: Peer review history

# Peer review: Decision Letter 0

**DOI:** 10.1371/journal.pone.0347588.r001

**Authors:** Wei Lang

2 Dec 2025

PONE-D-25-27496

Design of Urban Resilience Model in Emergency Situations

PLOS ONE

Dear Dr. Jiang,

Thank you for submitting your manuscript to PLOS ONE. After careful consideration, we feel that it has merit but does not fully meet PLOS ONE’s publication criteria as it currently stands. Therefore, we invite you to submit a revised version of the manuscript that addresses the points raised during the review process.

Please submit your revised manuscript by Jan 16 2026 11:59PM. If you will need more time than this to complete your revisions, please reply to this message or contact the journal office at plosone@plos.org. When you're ready to submit your revision, log on to https://www.editorialmanager.com/pone/ and select the 'Submissions Needing Revision' folder to locate your manuscript file.

Please include the following items when submitting your revised manuscript:

- A rebuttal letter that responds to each point raised by the academic editor and reviewer(s). You should upload this letter as a separate file labeled 'Response to Reviewers'.
- A marked-up copy of your manuscript that highlights changes made to the original version. You should upload this as a separate file labeled 'Revised Manuscript with Track Changes'.
- An unmarked version of your revised paper without tracked changes. You should upload this as a separate file labeled 'Manuscript'.

If you would like to make changes to your financial disclosure, please include your updated statement in your cover letter. Guidelines for resubmitting your figure files are available below the reviewer comments at the end of this letter.

If applicable, we recommend that you deposit your laboratory protocols in protocols.io to enhance the reproducibility of your results. Protocols.io assigns your protocol its own identifier (DOI) so that it can be cited independently in the future. For instructions see: https://journals.plos.org/plosone/s/submission-guidelines#loc-laboratory-protocols. Additionally, PLOS ONE offers an option for publishing peer-reviewed Lab Protocol articles, which describe protocols hosted on protocols.io. Read more information on sharing protocols at https://plos.org/protocols?utm_medium=editorial-email&utm_source=authorletters&utm_campaign=protocols.

We look forward to receiving your revised manuscript.

Kind regards,

Wei Lang, Ph.D.

Academic Editor

PLOS ONE

**Journal Requirements:**

1. When submitting your revision, we need you to address these additional requirements.

Please ensure that your manuscript meets PLOS ONE's style requirements, including those for file naming. The PLOS ONE style templates can be found at

https://journals.plos.org/plosone/s/file?id=wjVg/PLOSOne_formatting_sample_main_body.pdf and

https://journals.plos.org/plosone/s/file?id=ba62/PLOSOne_formatting_sample_title_authors_affiliations.pdf

2. We note that Figures 1, 5 and 6 in your submission contain map images which may be copyrighted. All PLOS content is published under the Creative Commons Attribution License (CC BY 4.0), which means that the manuscript, images, and Supporting Information files will be freely available online, and any third party is permitted to access, download, copy, distribute, and use these materials in any way, even commercially, with proper attribution. For these reasons, we cannot publish previously copyrighted maps or satellite images created using proprietary data, such as Google software (Google Maps, Street View, and Earth). For more information, see our copyright guidelines: http://journals.plos.org/plosone/s/licenses-and-copyright.

We require you to either present written permission from the copyright holder to publish these figures specifically under the CC BY 4.0 license, or remove the figures from your submission:

a. You may seek permission from the original copyright holder of Figures 1, 5 and 6 to publish the content specifically under the CC BY 4.0 license.

We recommend that you contact the original copyright holder with the Content Permission Form (http://journals.plos.org/plosone/s/file?id=7c09/content-permission-form.pdf) and the following text:

“I request permission for the open-access journal PLOS ONE to publish XXX under the Creative Commons Attribution License (CCAL) CC BY 4.0 (http://creativecommons.org/licenses/by/4.0/). Please be aware that this license allows unrestricted use and distribution, even commercially, by third parties. Please reply and provide explicit written permission to publish XXX under a CC BY license and complete the attached form.”

Please upload the completed Content Permission Form or other proof of granted permissions as an "Other" file with your submission.

In the figure caption of the copyrighted figure, please include the following text: “Reprinted from [ref] under a CC BY license, with permission from [name of publisher], original copyright [original copyright year].”

b. If you are unable to obtain permission from the original copyright holder to publish these figures under the CC BY 4.0 license or if the copyright holder’s requirements are incompatible with the CC BY 4.0 license, please either i) remove the figure or ii) supply a replacement figure that complies with the CC BY 4.0 license. Please check copyright information on all replacement figures and update the figure caption with source information. If applicable, please specify in the figure caption text when a figure is similar but not identical to the original image and is therefore for illustrative purposes only.

The following resources for replacing copyrighted map figures may be helpful:

USGS National Map Viewer (public domain): http://viewer.nationalmap.gov/viewer/

The Gateway to Astronaut Photography of Earth (public domain): http://eol.jsc.nasa.gov/sseop/clickmap/

Maps at the CIA (public domain): https://www.cia.gov/library/publications/the-world-factbook/index.html and https://www.cia.gov/library/publications/cia-maps-publications/index.html

NASA Earth Observatory (public domain): http://earthobservatory.nasa.gov/

Landsat: http://landsat.visibleearth.nasa.gov/

USGS EROS (Earth Resources Observatory and Science (EROS) Center) (public domain): http://eros.usgs.gov/#

Natural Earth (public domain): http://www.naturalearthdata.com/

3. We note that there is identifying data in the Supporting Information files. Due to the inclusion of these potentially identifying data, we have removed this file from your file inventory. Prior to sharing human research participant data, authors should consult with an ethics committee to ensure data are shared in accordance with participant consent and all applicable local laws.

Data sharing should never compromise participant privacy. It is therefore not appropriate to publicly share personally identifiable data on human research participants. The following are examples of data that should not be shared:

-Name, initials, physical address

-Ages more specific than whole numbers

-Internet protocol (IP) address

-Specific dates (birth dates, death dates, examination dates, etc.)

-Contact information such as phone number or email address

-Location data

-ID numbers that seem specific (long numbers, include initials, titled “Hospital ID”) rather than random (small numbers in numerical order)

Data that are not directly identifying may also be inappropriate to share, as in combination they can become identifying. For example, data collected from a small group of participants, vulnerable populations, or private groups should not be shared if they involve indirect identifiers (such as sex, ethnicity, location, etc.) that may risk the identification of study participants.

Additional guidance on preparing raw data for publication can be found in our Data Policy (https://journals.plos.org/plosone/s/data-availability#loc-human-research-participant-data-and-other-sensitive-data) and in the following article: http://www.bmj.com/content/340/bmj.c181.long.

Please remove or anonymize all personal information (<specific identifying information in file to be removed>), ensure that the data shared are in accordance with participant consent, and re-upload a fully anonymized data set. Please note that spreadsheet columns with personal information must be removed and not hidden as all hidden columns will appear in the published file.

4. Please include captions for your Supporting Information files at the end of your manuscript, and update any in-text citations to match accordingly. Please see our Supporting Information guidelines for more information: http://journals.plos.org/plosone/s/supporting-information.

5. If the reviewer comments include a recommendation to cite specific previously published works, please review and evaluate these publications to determine whether they are relevant and should be cited. There is no requirement to cite these works unless the editor has indicated otherwise.

[Note: HTML markup is below. Please do not edit.]

Reviewers' comments:

Reviewer's Responses to Questions

**Comments to the Author**

1. Is the manuscript technically sound, and do the data support the conclusions?

The manuscript must describe a technically sound piece of scientific research with data that supports the conclusions. Experiments must have been conducted rigorously, with appropriate controls, replication, and sample sizes. The conclusions must be drawn appropriately based on the data presented.

Reviewer #1: Yes

**********

2. Has the statistical analysis been performed appropriately and rigorously?

Reviewer #1: Yes

**********

3. Have the authors made all data underlying the findings in their manuscript fully available?

The PLOS Data policy requires authors to make all data underlying the findings described in their manuscript fully available without restriction, with rare exception (please refer to the Data Availability Statement in the manuscript PDF file). The data should be provided as part of the manuscript or its supporting information, or deposited to a public repository. For example, in addition to summary statistics, the data points behind means, medians and variance measures should be available. If there are restrictions on publicly sharing data—e.g. participant privacy or use of data from a third party—those must be specified.

Reviewer #1: Yes

**********

4. Is the manuscript presented in an intelligible fashion and written in standard English?

PLOS ONE does not copyedit accepted manuscripts, so the language in submitted articles must be clear, correct, and unambiguous. Any typographical or grammatical errors should be corrected at revision, so please note any specific errors here.

Reviewer #1: Yes

**********

5. Review Comments to the Author

Please use the space provided to explain your answers to the questions above. You may also include additional comments for the author, including concerns about dual publication, research ethics, or publication ethics. (Please upload your review as an attachment if it exceeds 20,000 characters)

**Reviewer #1:** This study proposes an urban resilience assessment framework for natural-disaster emergencies using housing, infrastructure, GDP, and population data from 25 districts in Changchun and Hohhot. Three indicators—emergency response speed, resource allocation efficiency, and per-capita facility density—are evaluated using AHP to determine their weights, and TOPSIS to compare the resilience levels of the two cities. The study identifies their major weaknesses, recommends short- and long-term investment strategies, and examines the strengths and limitations of existing resilience models through consistency and sensitivity analysis.

The research topic is interesting ; however, the manuscript contains several issues, which I have outlined in the following comments.

1. Please clarify the full term of the abbreviation “GDP” in the abstract.

2. In the introduction, when discussing prior studies, please provide more detailed information regarding their evaluation methods, datasets, and the strengths and limitations of their approaches. This will help situate your work more clearly within the existing literature.

3. Have any previous studies assessed urban resilience using POI (Point of Interest) data? If such work exists, it should be cited and compared with your approach.

4. The manuscript gives limited attention to related work and currently lacks a dedicated literature review section. Including a structured and comprehensive related-work section would significantly strengthen the theoretical grounding of the study.

5. Please elaborate on the key components and parameters traditionally used to measure urban resilience in the literature. How do the indicators you selected—such as parking fees or housing prices—fit within the established conceptual frameworks of urban resilience?

6. There are numerous methodological options for problems similar to yours. Please justify why AHP and TOPSIS were specifically chosen and explain why alternative methods were not considered or discussed.

7. The practical implications of your findings should be articulated more clearly. For instance, how can the results contribute to improving future decision-making or resource management in urban planning? These points should be explicitly addressed in the conclusion.

**********

6. PLOS authors have the option to publish the peer review history of their article (what does this mean?). If published, this will include your full peer review and any attached files.

If you choose “no”, your identity will remain anonymous but your review may still be made public.

**Do you want your identity to be public for this peer review?** For information about this choice, including consent withdrawal, please see our Privacy Policy.

Reviewer #1: No

**********

[NOTE: If reviewer comments were submitted as an attachment file, they will be attached to this email and accessible via the submission site. Please log into your account, locate the manuscript record, and check for the action link "View Attachments". If this link does not appear, there are no attachment files.]

To ensure your figures meet our technical requirements, please review our figure guidelines: https://journals.plos.org/plosone/s/figures

You may also use PLOS’s free figure tool, NAAS, to help you prepare publication quality figures: https://journals.plos.org/plosone/s/figures#loc-tools-for-figure-preparation.

NAAS will assess whether your figures meet our technical requirements by comparing each figure against our figure specifications.

# Author comment: Author response to Decision Letter 1

**DOI:** 10.1371/journal.pone.0347588.r002

26 Jan 2026

1. Please clarify the full term of the abbreviation “GDP” in the abstract.

Thank you for pointing this out. The abbreviation “GDP” stands for Gross Domestic Product. It is a key economic indicator that measures the total value of goods and services produced over a specific time period. In our study, GDP data were used as one of the factors to assess the economic resilience of cities, alongside other indicators such as population density and infrastructure levels. We have now explicitly defined “GDP” in the abstract to avoid any confusion.

2. In the introduction, when discussing prior studies, please provide more detailed information regarding their evaluation methods, datasets, and the strengths and limitations of their approaches. This will help situate your work more clearly within the existing literature.

We appreciate your suggestion to provide more detailed information on prior studies. We have revised the introduction to include more comprehensive descriptions of the evaluation methods, datasets, and strengths and limitations of previous research. For example, we have expanded our discussion on the work of Beheshtian et al. (2018), who used climatic data and energy consumption metrics to assess transportation resilience in New York City. Their study highlighted the importance of integrating multi-sector data but faced limitations in terms of spatial resolution and real-time data integration. Similarly, we have added more details on other studies, such as those by Elmqvist et al. (2019) and Meerow et al. (2016), to provide a clearer context for our work within the existing literature.

3. Have any previous studies assessed urban resilience using POI (Point of Interest) data? If such work exists, it should be cited and compared with your approach.

Thank you for raising this important point. Indeed, there have been several studies that have utilized Point of Interest (POI) data to assess urban resilience. For instance, Chen et al. (2024) used social media data linked to POI information to evaluate urban vibrancy during the COVID-19 pandemic. Another example is the work by Zhong et al. (2025), who integrated POI data with geospatial analysis to assess green space accessibility in the Yangtze River Delta. We have now cited these studies and compared them with our approach, highlighting how our work builds on previous efforts while introducing new dimensions such as the integration of economic parameters and comprehensive investment planning.

4. The manuscript gives limited attention to related work and currently lacks a dedicated literature review section. Including a structured and comprehensive related-work section would significantly strengthen the theoretical grounding of the study.

We agree that a dedicated literature review section would significantly enhance the theoretical grounding of our study. We have therefore added a new section titled “Related Work,” where we systematically review the existing literature on urban resilience. This section provides an overview of key concepts, methodologies, and findings from previous studies. It also discusses the gaps in current research and how our study addresses these gaps. We believe this addition will provide readers with a more comprehensive understanding of the context and significance of our work.

5. Please elaborate on the key components and parameters traditionally used to measure urban resilience in the literature. How do the indicators you selected—such as parking fees or housing prices—fit within the established conceptual frameworks of urban resilience?

Existing studies typically treat POIs as simple count indicators for single hazards or single functions, assign weights subjectively to produce heat maps, lack coupling with economic parameters, and do not answer the question “with a limited budget, where should investment go first?” In contrast, this study incorporates fifteen POI categories into a three dimensional “distance–density–economy” matrix, derives emergency scenario weights via AHP, and uses TOPSIS to convert each district’s distance to the ideal solution into an investment gap ratio. Short and long term investments are then allocated proportionally, yielding a concrete roster of facilities and investment amounts (in units of 100 million yuan), thus filling the gap between having indicators and having an implementable plan.

Traditional frameworks emphasize five pillars—social, economic, environmental, physical, and institutional—but often stall in practice because indicators are difficult to quantify, weights are hard to reach consensus on, and results are hard to translate into actionable investments. In our framework, for example, housing prices and parking fees are modeled as dual “economic–physical” factors: housing price deviation captures affordability, while parking price differentials reflect transportation structure. These two factors jointly adjust per capita facility density scores, allowing price signals and spatial signals to interact within the same vector. After AHP–TOPSIS integration, districts with high housing prices and low facility density naturally shift toward the negative ideal and receive higher investment priority, thereby converting the abstract objectives of the five pillars into expressions that are actionable, budgetable, and trackable.

6. There are numerous methodological options for problems similar to yours. Please justify why AHP and TOPSIS were specifically chosen and explain why alternative methods were not considered or discussed.

We appreciate the reviewer’s request for clarification. The problem addressed in our study is a multi‑criteria decision problem that combines qualitative expert judgments with quantitative indicators; therefore we selected methods that together (1) elicit reliable criterion weights from expert judgments and (2) produce an interpretable ranking of alternatives.

AHP was chosen because it supports hierarchical structuring of complex criteria, converts pairwise subjective judgments into numerical weights, and provides a consistency ratio to check the reliability of those judgments. This makes AHP particularly suitable when expert preferences are important and need formal consistency testing.

TOPSIS was selected because it ranks alternatives by their distance to an ideal and anti‑ideal solution, which is intuitive, computationally efficient, and easily interpretable by practitioners and stakeholders. TOPSIS integrates naturally with externally derived weights (e.g., from AHP) and yields transparent ranking results.

The combined AHP+TOPSIS workflow leverages complementary strengths: AHP provides defensible, consistency‑checked weights; TOPSIS delivers a clear, reproducible ranking based on those weights. This combination is widely used in applied engineering, management, and policy studies for its balance of rigor and interpretability.

We did consider alternative MCDM methods (e.g., entropy weighting, VIKOR, PROMETHEE, MAUT, fuzzy AHP). These were not adopted for the following practical reasons: entropy weighting relies solely on objective data and does not capture expert preference; VIKOR focuses on compromise solutions which were not the objective here; PROMETHEE requires specifying preference functions that reduce transparency for our audience; MAUT demands detailed utility elicitation and extensive data; fuzzy approaches increase modelling complexity and reduce reproducibility given our data and time constraints. Thus the alternatives were excluded based on suitability, data requirements, complexity, and interpretability considerations.

The selection of AHP and TOPSIS was not driven by “methodological preference,” but rather a rational choice constrained by data attributes, decision objectives, and budgetary context. This paper addresses a typical multi-criteria, low-sample, high-stakes public investment decision: “Should cities invest in infrastructure during sudden extreme weather events, and where should they prioritize?” With 25 districts/counties, 15 facility types, and 3 major indicator dimensions, the sample size is far too small to support the training-validation split required for machine learning. Additionally, the indicators feature mixed units (distance-density-property prices), and the final output must be an interpretable ranking of “who gets priority and how much funding,” not merely predicted probabilities. AHP's value in this scenario lies in explicitly structuring “expert judgment”: it translates the difficult-to-quantify policy preference of “preserving lives and maintaining operations during emergencies” into a testable weight vector through pairwise comparisons. The consistency ratio of 0.046 < 0.1 also demonstrates logical coherence in decision-making. More importantly, it allows the subjective amplification of “emergency response speed” to 52.8%, aligning with the disaster management consensus that “time is paramount”—a normative value that purely data-driven methods cannot a priori incorporate. TOPSIS then seamlessly integrates the weights generated by AHP, quantifying the ideal utopia (“shortest distance-highest density-most affordable housing”) and the worst nightmare. By calculating Euclidean distances, it determines each district's relative position to the “ideal solution,” naturally producing a rankable proximity index. This index can be linearly mapped to fiscal gaps, directly translating into specific investment allocations—60% for short-term and 40% for long-term—enabling seamless integration between ranking and budgeting. In contrast, while entropy weighting or principal component analysis are objective, they excessively amplify high-variance indicators like “housing price differences,” drowning out critical low-variance factors like ‘distance’; DEA excels at evaluating “efficiency frontiers” but fails to handle the cross-impact of price signals and spatial signals; structural equation or factor analysis requires large-sample survey data, whereas this study only has aggregated district/county indicators; Machine learning methods (Random Forest, XGBoost) can capture nonlinearity, but with small samples and few variables, they are prone to overfitting. Moreover, their “black box” outputs make it difficult to justify to finance and audit departments why District A receives 3 billion yuan while District B gets only 500 million. Therefore, the authors explicitly state in the methodology section: Given limited data scale, mandatory decision transparency, and the requirement for one-to-one correspondence between weights and fiscal items, the AHP-TOPSIS combination offers the shortest path to an “explainable, verifiable, and implementable” solution. Consequently, other data-driven or black-box methods were excluded from consideration.

7. The practical implications of your findings should be articulated more clearly. For instance, how can the results contribute to improving future decision-making or resource management in urban planning? These points should be explicitly addressed in the conclusion.

We have revised the discussion to more explicitly address the practical implications of our findings. Our study provides actionable insights for urban planners and policymakers by identifying key areas for investment and improvement in urban resilience. For example, our results highlight the need for enhanced public facilities and financial services in Changchun, and improved transportation infrastructure in Hohhot. We have also discussed how our findings can inform future decision-making in urban planning, such as through fiscal programming, land-use regulation, and infrastructure procurement. By translating our model outputs into specific policy instruments, we aim to bridge the gap between evaluation and execution, ensuring that our research directly contributes to practical urban planning efforts.

Attachment Submitted filename: Response to Reviewers.docx

# Peer review: Decision Letter 1

**DOI:** 10.1371/journal.pone.0347588.r003

**Authors:** Wei Lang

5 Apr 2026

Design of Urban Resilience Model in Emergency Situations

PONE-D-25-27496R1

Dear Dr. Jiang,

We’re pleased to inform you that your manuscript has been judged scientifically suitable for publication and will be formally accepted for publication once it meets all outstanding technical requirements.

Within one week, you’ll receive an e-mail detailing the required amendments. When these have been addressed, you’ll receive a formal acceptance letter and your manuscript will be scheduled for publication.

An invoice will be generated when your article is formally accepted. Please note, if your institution has a publishing partnership with PLOS and your article meets the relevant criteria, all or part of your publication costs will be covered. Please make sure your user information is up-to-date by logging into Editorial Manager at Editorial Manager® and clicking the ‘Update My Information' link at the top of the page. For questions related to billing, please contact billing support.

If your institution or institutions have a press office, please notify them about your upcoming paper to help maximize its impact. If they’ll be preparing press materials, please inform our press team as soon as possible -- no later than 48 hours after receiving the formal acceptance. Your manuscript will remain under strict press embargo until 2 pm Eastern Time on the date of publication. For more information, please contact onepress@plos.org.

Kind regards,

Wei Lang, Ph.D.

Academic Editor

PLOS One

Additional Editor Comments (optional):

Reviewers' comments:

Reviewer's Responses to Questions

**Comments to the Author**

1. If the authors have adequately addressed your comments raised in a previous round of review and you feel that this manuscript is now acceptable for publication, you may indicate that here to bypass the “Comments to the Author” section, enter your conflict of interest statement in the “Confidential to Editor” section, and submit your "Accept" recommendation.

Reviewer #2: (No Response)

**********

2. Is the manuscript technically sound, and do the data support the conclusions?

The manuscript must describe a technically sound piece of scientific research with data that supports the conclusions. Experiments must have been conducted rigorously, with appropriate controls, replication, and sample sizes. The conclusions must be drawn appropriately based on the data presented.

Reviewer #2: (No Response)

**********

3. Has the statistical analysis been performed appropriately and rigorously?

Reviewer #2: (No Response)

**********

4. Have the authors made all data underlying the findings in their manuscript fully available?

The PLOS Data policy requires authors to make all data underlying the findings described in their manuscript fully available without restriction, with rare exception (please refer to the Data Availability Statement in the manuscript PDF file). The data should be provided as part of the manuscript or its supporting information, or deposited to a public repository. For example, in addition to summary statistics, the data points behind means, medians and variance measures should be available. If there are restrictions on publicly sharing data—e.g. participant privacy or use of data from a third party—those must be specified.

Reviewer #2: (No Response)

**********

5. Is the manuscript presented in an intelligible fashion and written in standard English?

PLOS ONE does not copyedit accepted manuscripts, so the language in submitted articles must be clear, correct, and unambiguous. Any typographical or grammatical errors should be corrected at revision, so please note any specific errors here.

Reviewer #2: (No Response)

**********

6. Review Comments to the Author

Please use the space provided to explain your answers to the questions above. You may also include additional comments for the author, including concerns about dual publication, research ethics, or publication ethics. (Please upload your review as an attachment if it exceeds 20,000 characters)

Reviewer #2: (No Response)

**********

7. PLOS authors have the option to publish the peer review history of their article (what does this mean?). If published, this will include your full peer review and any attached files.

If you choose “no”, your identity will remain anonymous but your review may still be made public.

**Do you want your identity to be public for this peer review?** For information about this choice, including consent withdrawal, please see our Privacy Policy.

Reviewer #2: No

**********

# Editor report: Acceptance letter

**DOI:** 10.1371/journal.pone.0347588.r004

**Authors:** Wei Lang

PONE-D-25-27496R1

PLOS One

Dear Dr. Jiang,

I'm pleased to inform you that your manuscript has been deemed suitable for publication in PLOS One. Congratulations! Your manuscript is now being handed over to our production team.

At this stage, our production department will prepare your paper for publication. This includes ensuring the following:

* All references, tables, and figures are properly cited

* All relevant supporting information is included in the manuscript submission,

* There are no issues that prevent the paper from being properly typeset

You will receive further instructions from the production team, including instructions on how to review your proof when it is ready. Please keep in mind that we are working through a large volume of accepted articles, so please give us a few days to review your paper and let you know the next and final steps.

Lastly, if your institution or institutions have a press office, please let them know about your upcoming paper now to help maximize its impact. If they'll be preparing press materials, please inform our press team within the next 48 hours. Your manuscript will remain under strict press embargo until 2 pm Eastern Time on the date of publication. For more information, please contact onepress@plos.org.

You will receive an invoice from PLOS for your publication fee after your manuscript has reached the completed accept phase. If you receive an email requesting payment before acceptance or for any other service, this may be a phishing scheme. Learn how to identify phishing emails and protect your accounts at https://explore.plos.org/phishing.

If we can help with anything else, please email us at customercare@plos.org.

Thank you for submitting your work to PLOS ONE and supporting open access.

Kind regards,

PLOS ONE Editorial Office Staff

on behalf of

Dr. Wei Lang

Academic Editor

PLOS One
